# Contact-Piezoelectric Bi-Catalysis of an Electrospun ZnO@PVDF Composite Membrane for Dye Decomposition

**DOI:** 10.3390/molecules27238579

**Published:** 2022-12-05

**Authors:** Buwen Jiang, Xiaoxuan Xue, Zuxiang Mu, Haoyuan Zhang, Feng Li, Kai Liu, Wenqian Wang, Yongfei Zhang, Wenhui Li, Chao Yang, Kewei Zhang

**Affiliations:** College of Material Science and Engineering, Qingdao University, Qingdao 266071, China

**Keywords:** contact-electro-catalysis, piezoelectric catalysis, ZnO@PVDF composite membrane, methyl orange

## Abstract

The treatment of organic pollutants in wastewater is becoming a great challenge for social development. Herein, a novel contact-piezoelectric bi-catalysis of a ZnO@ PVDF composite membrane was prepared by electrospinning technology. The obtained ZnO@PVDF composite membranes is superior to the pure PVDF membrane in decomposing methyl orange (MO) under ultrasonication at room temperature, which is mainly attributed to the synergy effect of the contact-electro-catalysis of dielectric PVDF, as well as the piezoelectric catalysis of tetrapodal ZnO and the β-phase of PVDF. The heterostructure of the piezoelectric-ZnO@dielectric-PVDF composite is beneficial in reducing the electron/hole pair recombination. As compared to the pure PVDF membrane, the catalytic degradation efficiency of the ZnO@PVDF composite membrane was improved by 444.23% under ultrasonication. Moreover, the reusability and stability of the composite membrane are comparable to those of the traditional powdered catalyst. This work offers a promising strategy for improving the pollutant degradation by combining contact-electro-catalysis with piezoelectric catalysis.

## 1. Introduction

In recent years, the treatment of wastewater has become a great challenge for social development [1]. Pollutants in water include organic and inorganic pollutants. Compared with inorganic pollutants, organic pollutants are difficult to decompose because of their high concentration and complex components. About 15% of dyes produced in industrial processes are discharged into the global environment every year, which leads to adverse effects on human health. Consequently, it is urgent to find an effective method to solve this issue [2].

At present, there are many methods to treat organic dyes, such as photocatalytic degradation [3], biodegradation [4], redox degradation [5], and mechanochemistry catalytic degradation [6], which have all been developed and improved, to a certain degree. Among these, the mechanochemistry catalytic degradation as one kind physic catalysis exhibits special characteristics because it does not involve complicated chemical reactions and has the potential to use environmental mechanical energy. Ultrasonic catalytic degradation has recently attracted particular attention due to its mineralization of various organic pollutants, easy operation, safety, and environmental benefits [7,8,9,10,11,12,13].

Recently, studies on the mechanism of contact charging at the liquid-solid interface, called contact-electro-catalysis, have been reported, indicating that electrons play a dominant role in charge transfer at the interface. The frequent contact-separation cycles at the surface of dielectric powder induce the exchange and transfer of electrons to different substrates to form reactive oxygen species to degrade organic compounds [14,15,16]. This mechanism occurs in dielectric materials such as perfluorinated ethylene propylene copolymer (FEP), Teflon, nylon-6,6, and rubber. Contact-electro-catalysis has been proved to be a feasible new catalytic mechanism [16,17,18,19]. It greatly broadens the selection range of catalysts and makes it possible to design more abundant catalytic systems. However, similar to the triboelectric mechanocatalytic efficiency (<4.3%), the contact-electro-catalysis efficiency is very low, which limits its practical applications [20].

Regarding another kind of mechanochemistry piezoelectric effect, which is also used to degrade pollutants in wastewater, zinc oxide (ZnO) is one of popular piezoelectric materials, and its excellent piezoelectric property is favored by many researchers [21,22]. ZnO has a direct broadband gap close to 3.37 eV, and a high excitation binding energy of 60 meV in the near-ultraviolet wavelength environment at room temperature [23]. The piezoelectric effect exhibits an energy conversion efficiency of above 35%, as reported by the authors of [24].

Ultrasonication is an external technology used to provide the mechanical vibration wave to realize the mechanochemical process. Ultrasonic wave propagation in solutions causes the formation of cavitation bubbles (CB). It is assumed that the collapse of CB leads to frequent contact charging at the solid–water interface, resulting in contact-electro-catalysis [16], while the spontaneous polarization of piezoelectric material, after capturing vibration energy, leads to inherent free holes and electron separation. The separated current carriers are aggregated on the surface of the piezoelectric catalyst and perform a series of redox reactions with the surrounding reactants, which can be used to degrade organic pollutants, decompose water to produce hydrogen, and remove bacteria [25,26,27]. However, the piezoelectric catalytic materials are generally powders, which are not very convenient to recycle. Polyvinylidene fluoride(PVDF) is expected to be an ideal carrier for the above catalysis due to its stable chemical property, flexibility, and potential piezoelectric performance sensitivity [28].

Electrospinning is a spinning technique in which electrostatic force is used to produce finer fibers (with a diameter from nanometers to microns) from a polymer solution or melt, and then the electrospinning membrane is provided with a larger surface area than that obtained by conventional spinning [29]. The larger the contact area between catalyst and reactant, the better the catalytic effect. The PVDF membrane, with a larger specific surface area prepared by electrospinning, is anticipated to be more effective as a degradation catalysis than traditional PVDF membranes in other forms.

In this work, the ZnO@PVDF composite membranes were prepared by electrospinning technology, and the degradation efficiency of MO solution using the composite membrane was studied at room temperature. The tetrapodal ZnO was chosen as the piezoelectric element because it is conducive to the formation of an embedded structure between the ZnO and PVDF fiber. SEM, BET, EDS, XRD, and TG were used to characterize the morphology, specific surface area, element distribution, crystal phase composition, and ZnO content of the ZnO@PVDF composite membrane, respectively. The MO solution was degraded with the above electrospinning membrane, and the degradation efficiency was detected using a UV absorption spectrum tester. The effects of ultrasonic time and MO concentration on degradation efficiency were detected, the reusability and stability of the membrane were explored, and the composite catalytic mechanism was proposed. Based on the ZnO@PVDF electrospinning composite membrane, the contact-electro-catalysis and piezoelectric catalysis were organically combined to further improve the efficiency of mechanochemistry catalysis. This work provides a new potential solution for the effective removal of organic dyes.

## 2. Results and Discussion

### 2.1. Characterization of the ZnO@PVDF Composite Membrane

**Figure 1:**
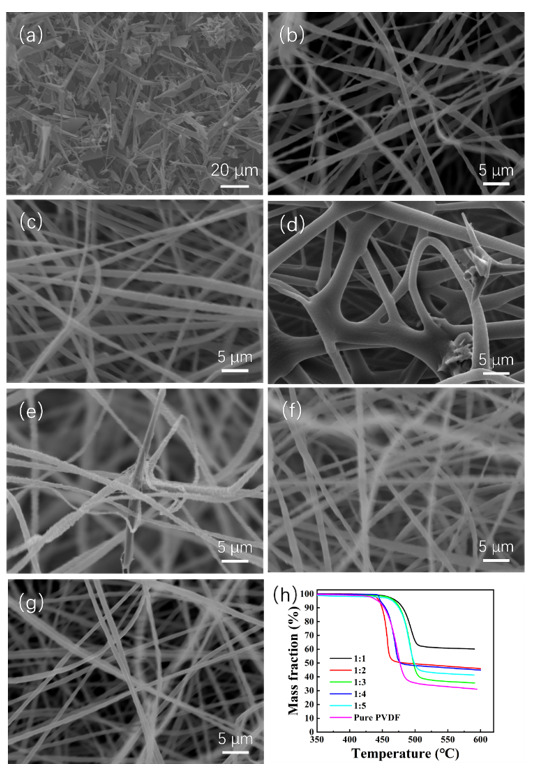
(**a**) SEM image of tetrapodal ZnO. (**b**–**f**) SEM images of the electrospinning ZnO@PVDF composite membranes with mass ratios of ZnO:PVDF = 1:1 (**b**), 1:2 (**c**), 1:3 (**d**), 1:4 (**e**), and 1:5 (**f**). (**g**) SEM image of the electrospinning pure PVDF membrane. (**h**) TG curves of the ZnO@PVDF composite membranes with different ZnO:PVDF mass ratios.

The morphology of the ZnO@PVDF composite membranes was characterized with SEM, as shown in Figure 1; the tetrapodal ZnO was chosen as the functional addition, and its size distribution is not uniform (Figure 1a). Through electrospinning technology, ZnO and PVDF were composited, and a three-dimensional (3D) network structure was built. The diameter of a single PVDF fiber is 1~3 μm, specifically, the diameter of sample 1:1, 1:2, 1:3, 1:4, 1:5, and pure PVDF is ~1.2 μm, ~1.5 μm, ~3.0 μm, ~1.1 μm, ~1.2 μm, and ~1.0 μm, respectively (Figure 1b–g). Compared with other ZnO@PVDF composite membranes, the pure PVDF membrane possessed the finest and smoothest morphology (Figure 1b). The gaps among the microscale fibers provide a large existing space for ZnO and a high specific surface area for water to make full contact with the solid surface, since full contact is necessary for efficient catalysis. From Figure 1c–g, it can be seen that the concentration of ZnO is decreasing, the surface of the fibers is roughing, and there are rich folds. We consider this to be attributed to the wrapped tetrapodal ZnO, which has been whipped to pieces. The chosen tetrapodal ZnO has four needles, and it is broken during the long-time magnetic stirring process before spinning, as well as through the needle during the electrospinning process. Consequently, most ZnO are evenly dispersed as particles or short fibers into the electrospinning membrane. However, for the whole tetrapodal ZnO, as shown in Figure 1d,e, it is locked into the 3D network of electrospinning fibers, and in turn, it acts as a link among the fibers.

Simultaneously, TG is used to determine the body content of ZnO in the ZnO@PVDF composite membranes (Figure 1h). The residual mass fraction after the pyrolysis of pure PVDF in nitrogen is 31.10%, which is the residual carbon. Then, when we calculate the ZnO content of the corresponding composite membranes, the residual carbon content should be subtracted from the pyrolysis product. As shown in Figure 1a, ZnO particles present multi-sized morphology, and the ZnO content of the five samples likely matches with that of the raw electrospinning solution. The specific content is 48.31%, 32.33%, 12.62%, 30.69%, and 24.73%, respectively, from sample 1:1 to 1:5. Different ZnO content affects the conductivity of the electrospinning fiber, so the electrospinning voltage was adjusted, which resulted in different diameters for a single composite fiber. Theoretically, the ZnO content will be less than that of the initial spinning solution because of material loss during the electrospinning process. For sample 1:3, the diameter is bigger and the gap among the single fibers is narrow, so less tetrapodal ZnO is locked in the composite membrane, and the ZnO content is lower than the content of the initial electrospinning solution.

**Figure 2:**
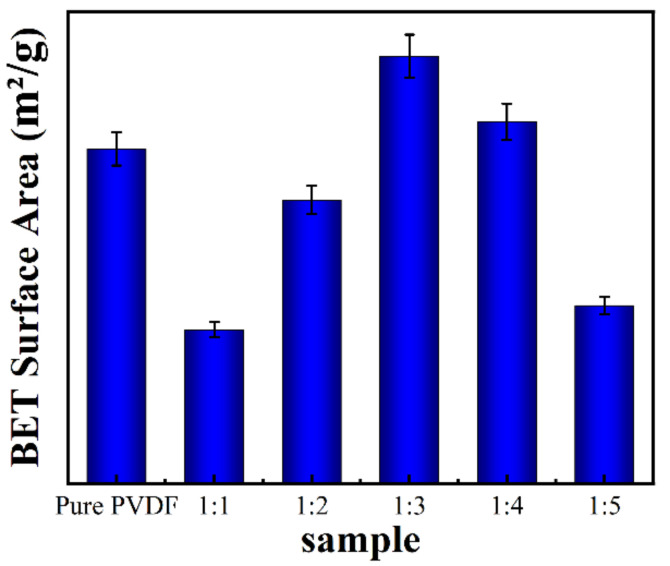
BET surface area of the electrospinning pure PVDF membrane and the ZnO@PVDF composite membranes with mass ratios of ZnO:PVDF = 1:1, 1:2, 1:3, 1:4, and 1:5, respectively.

In order to detect the specific surface area of the samples, the structure morphology was characterized by BET technology (Figure 2). Because the density of ZnO is greater than that of the PVDF, the addition of ZnO decreases the looseness of the 3D structure, except for sample 1:3, which presents the largest specific surface area attributed to the enhanced mechanical property of the ZnO@PVDF fibers. This means that with an appropriate ZnO content, the mechanical property becomes optimal for the ZnO@PVDF composite materials. A higher ZnO content is not necessarily better. Stronger single composite fibers can sustain a looser 3D structure of a composite membrane. For a similar reason, sample 1:4 also presents a little larger specific surface area than does the pure PVDF, as also seen in sample 1:3, while other samples show a smaller specific surface area than does the pure PVDF membrane, presenting different values with different ZnO content.

**Figure 3:**
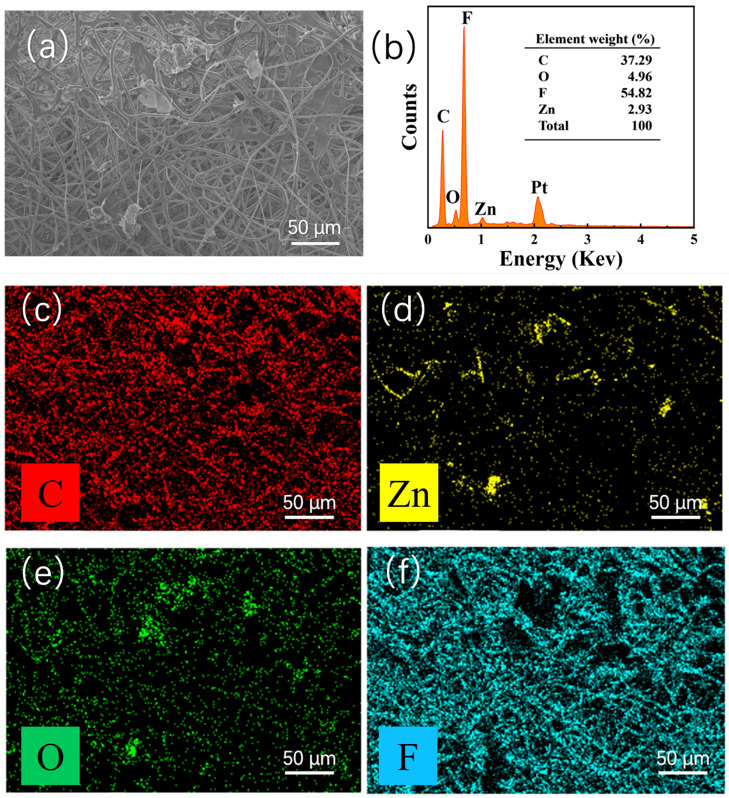
EDS of the ZnO@PVDF composite membrane (ZnO/PVDF = 1:3, *w*/*w*). (**a**) SEM image of the overview. (**b**) Elemental content analysis. (**c**–**f**) Elemental mapping, showing C, Zn, O, and F distributions.

To further analyze the composition of the electrospinning ZnO@PVDF composite membranes, we selected the composite membrane (ZnO/PVDF = 1:3, *w*/*w*) as a representative to analyze the elemental composition using an energy dispersive spectrometer (Figure 3). The elements and content on the surface of the sample shown in Figure 3a are shown in Figure 3b; there are four elements of C, O, F, and Zn observed on the sample, not including Pt, which was introduced when spraying with the conductive element before testing. The peaks prove the existence of Zn and O elements in PVDF. It is noted that the C and F elements account for the highest proportion of elements on the sample surface, which were 37.29% and 54.82%, respectively, because the body polymer PVDF is made of hydrocarbon compounds, while the Zn and O elements on the sample surface are 2.93% and 4.96%, respectively. The O content is higher than the Zn content, resulting from the absorbed oxygen in air on the surface of the ZnO@PVDF composite membranes. In addition, Figure 3c–f uses different colors to represent different elements; the yellow dots represent the Zn element distributed on the membrane, and the position of the green dots, representing the O element, corresponds to the position of the Zn element, which indicates the existence of ZnO. In addition, the data show that the surface ZnO content of the composite membrane does not match with the body content (ZnO/PVDF = 1:3, *w*/*w*). This indicates that most of the ZnO particles are embedded into the PVDF fibers.

**Figure 4:**
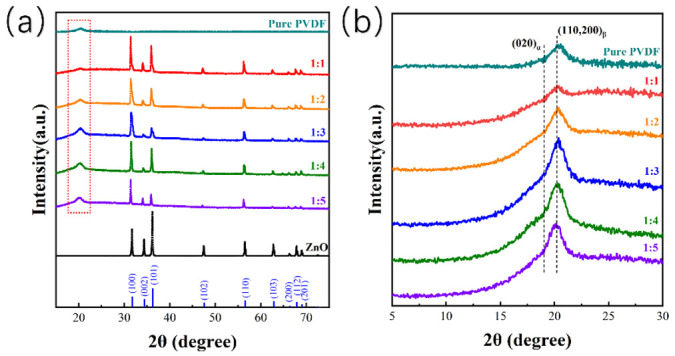
XRD patterns used to investigate the crystal phase composition. (**a**) XRD patterns of the electrospinning pure PVDF membrane, and the tetrapodal ZnO and ZnO@PVDF composite membranes with mass ratios of ZnO:PVDF = 1:1, 1:2, 1:3, 1:4, and 1:5, respectively. (**b**) Enlarged view of the red dotted box in (**a**).

Different compositions lead to different phase compositions. The XRD technique was used to investigate the crystal phase composition of the samples (Figure 4). The crystal structure of the tetrapodal ZnO is nearly maintained in the ZnO@PVDF composite membranes, laying the foundation for piezoelectric catalysis (Figure 4a). Moreover, the addition of the ZnO induced more β-phase content into the PVDF (Figure 4b). Except for sample 1:1, all the ZnO@PVDF composite membranes present a higher intensity of the β200/110 diffraction peak at 2θ = 20.2° and a lower intensity of the α020 diffraction peak at 2θ = 19.02° compared with the electrospinning pure PVDF membrane. However, with the increase in ZnO content, the homogeneity of the composite material cannot be guaranteed, so the induced performance is not obvious, and the β-phase content did not increase obviously for sample 1:1. More organic-inorganic phase interfaces enhance the induced formation of more β-phase content of PVDF by ZnO, which is attributed to the piezoelectric catalysis of PVDF. The sample 1:3 possesses the highest β200/110 diffraction peak compared with the other samples. Thus, the contact-piezoelectric bi-catalysis is also embodied in the piezoelectric catalysis of the β-phase PVDF of the ZnO@PVDF composite membrane by compositing using electrospinning technology without the need for a complex polarization processes.

### 2.2. Contact-Piezoelectric Bi-Catalytic Performance

To detect the degradation efficiency of the ZnO@PVDF composite membranes, we tested the concentration of MO solution under different conditions using UV-Vis spectroscopy; this crucial testing method is in accordance with the Lambert–Beer theorem, as shown in Equation (1), to calculate the concentration of methyl orange solution after ultrasonication.

(1) A=kbc

where *A* is the absorption value of the sample measured by UV-Vis spectroscopy, *k* is the molar absorption coefficient, *b* is the inner diameter width of the cuvette, and *c* is the concentration of the solution to be measured. The optimal UV absorption wavelength of the MO solution is 463 nm, which represents the concentration of MO solution in this system [30]. 

**Figure 5:**
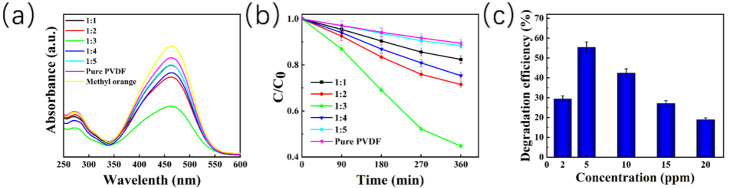
Degradation efficiency of 5 ppm MO solution by ultrasonic treatment using different ZnO@PVDF composite membranes. (**a**) UV-Vis spectra of the MO solutions after 360 min. (**b**) Decomposition of MO solution at different treatment times; C_0_ and C is the initial and instant concentration, respectively. (**c**) Catalytic degradation efficiency of the ZnO@PVDF composite membrane (ZnO/PVDF = 1:3, *w*/*w*) at different initial concentrations of MO solution.

The catalytic degradation efficiency of different ZnO@PVDF composite membranes with different mass ratios of ZnO/PVDF was also different. For the pure PVDF electrospinning membrane, the MO degradation is due to the contact-electro-catalysis performance, as shown in Figure 5, and the degradation efficiency is only 10.60%. When ZnO particles were added, the piezoelectric effect was enhanced. We explored the degradation efficiency of the composite membranes with the mass ratio of ZnO/PVDF of 1:1, 1:2, 1:3, 1:4, and 1:5, respectively, on MO dye. All samples were placed in 5ppm MO solution under ultrasonication for 360 min at room temperature. The five degradation efficiencies from 1:1 to 1:5 are 17.61%, 28.53%, 55.29%, 24.68%, and 11.60%, respectively, and all the degradation efficiencies are higher than that of the pure PVDF electrospinning membrane. This indicates that the effect of adding ZnO, along with the composite membrane (ZnO/PVDF = 1:3, *w*/*w*), presents the highest catalytic efficiency. The decomposition efficiency for the MO solution increases with the increase in reaction time in the process of catalytic decomposition. However, the rate of increase is the fastest from 90 min to 180 min, and the degradation efficiency increases slowly near the end of the reaction (360 min), taking the MO concentration of 5 ppm as representative (Figure 5b). In order to detect the effect of MO concentration on the decomposition efficiency, we selected the ZnO@PVDF composite membrane (ZnO/PVDF = 1:3, *w*/*w*) as a representative and placed this sample in MO solution with concentrations of 2 ppm, 5 ppm, 10 ppm, 15 ppm, and 20 ppm, respectively (Figure 5c). The MO solutions, along with the composite membranes, were ultrasonicated for 360 min under the same conditions (Figure 3d). The degradation efficiency of the ZnO@PVDF composite membrane (ZnO/PVDF = 1:3, *w*/*w*), with different MO concentration from 2 ppm to 20 ppm, was 29.21%, 55.29%, 42.35%, 27.05%, and 19.52%, respectively. The results indicate that the ZnO@PVDF composite membrane (ZnO/PVDF = 1:3, *w*/*w*) expresses the highest catalysis efficiency for the 5ppm MO solution; this may be related to the proportion of effective collisions at different concentrations, as well as the easy annihilation of free radicals in cramped, small spaces in the MO solution with high concentrations.

Theoretically, the higher the ZnO content, the higher the catalytic degradation efficiency. However, when the ZnO@PVDF composite membrane (ZnO/PVDF = 1:3, *w*/*w*) has the highest catalytic performance, the MO concentration can be reduced to 45% after 360 min under ultrasonication, although the ZnO content is only 12.62%. There are two reasons for this: one is that due to the large specific surface area, the full contact of MO with the ZnO@PVDF composite membrane is essential for an efficient catalyst comprised of contact-electro-catalysis and piezoelectric catalysis. Under ultrasonication, the full contact shifts to more reactive sites, and the degradation efficiency is clearly increased. Thus, sample 1:3 presents the highest efficiency because it possesses the largest specific surface area, attributed to the enhanced mechanical property of the ZnO@PVDF single fibers with an optimal ZnO content. The other reason is that the largest diameter of the single fibers prefer to embed more ZnO particles or ZnO short bars from the broken tetrapodal ZnO, which results in the full contact of ZnO and PVDF, leading to more organic–inorganic phase interfaces compared with those in other samples. This enhances the induced formation of more β-phase content of PVDF by ZnO, which is mainly attributed to the piezoelectric catalysis of PVDF. Consequently, part of the inefficient contact-electro-catalysis transfers to the efficient piezoelectric catalysis, which results in the increased efficiency of the degradation of MO solution.

### 2.3. Reusability and Stability of the ZnO@PVDF Composite Membrane

**Figure 6:**
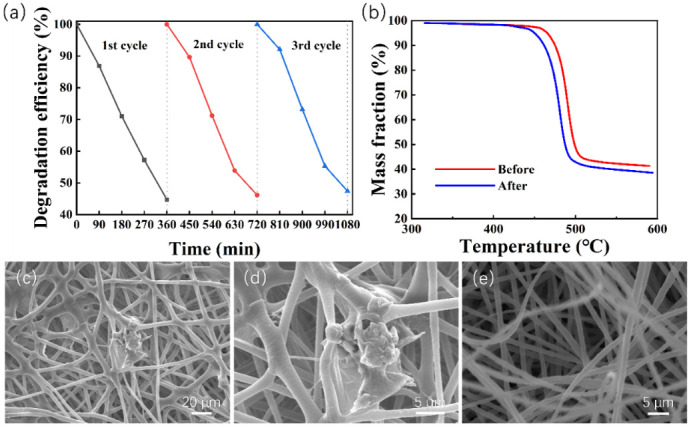
Reusability and stability of the ZnO@PVDF composite membrane (ZnO/PVDF = 1:3, *w*/*w*). (**a**) Three degradation cycles of MO solution using the composite membrane (ZnO/PVDF = 1:3, *w*/*w*) under ultrasonication at room temperature. (**b**) TG curves of the ZnO@PVDF composite membrane before and after ultrasonic treatment for three cycles. (**c**–**e**) SEM images of the composite membrane after three-cycle degradation.

In terms of practical application, reusability and stability are of primary importance regarding the performance of a catalyst. To evaluate the reusability and stability of the ZnO@PVDF composite membrane, the composite membrane was recycled and used for MO degradation under the same experimental conditions (MO solution of 5 ppm, 80 kHz, 700 W, room temperature). We choose the ZnO@PVDF composite membrane (ZnO/PVDF = 1:3, *w*/*w*) as the representative, and performed the test using three catalytic cycles; each cycle lasted 360 min, and the results are shown in Figure 6a. Compared with the first cycle, the catalytic efficiency decreased to 96.89% and 94.34% after the second and third cycle, respectively. The specific degradation efficiency was 55.29%, 53.59%, and 52.17% for the three cycles, respectively. It is obvious that the ZnO@PVDF composite membrane demonstrated excellent catalytic performance, and the catalyst maintained a high performance for MO decomposition after the third reaction of catalyst recycling. Only a 3.12% decrease in degradation efficiency was observed, suggesting that the ZnO@PVDF composite membrane possesses high reusability towards MO degradation. In addition, the TG and SEM of the ZnO@PVDF composite membrane before and after the three cycles are shown in Figure 6b–e to detect the change of composition and morphology, respectively, in the ZnO@PVDF composite membrane. The TG curves show that the mass fraction of ZnO before and after ultrasonication is 12.62% and 9.84%, respectively (Figure 6b). The decrease in ZnO content is 2.78%, which indicates that ultrasonication caused the ZnO particles to fall off the membrane surface. With the catalysis processing, there are no surface particles to be stripped, and thus, the composition tends to be stable. Compared with the original membrane, the 3D network has been entirely maintained, the needles or tips of ZnO on the membrane surface disappeared, the ZnO particles became a little smoother, and the gaps in the composite membrane expanded, becoming a little larger (Figure 1 and Figure 6c–e). Consequently, the structural change is very small, and the composition is stable; these two aspects confirm the reusability and stability of the ZnO@PVDF composite membranes.

### 2.4. Mechanism of Contact-Piezoelectric Bi-Catalysis Process

This work is based on the use of the electrospinning ZnO@PVDF composite membrane to explore the high degradation efficiency of MO solution under ultrasonication. Under the contact-piezoelectric bi-catalysis, the as-prepared ZnO@ PVDF composite membrane generates a large number of electrons at the interface of PVDF-water and ZnO-water. As a dielectric material, PVDF can store electrons generated by the contact-electric and piezoelectric effect in a timely manner, prolonging the time of the electron and hole existence on these two types of interfaces, thus ensuring the long-term existence of free radicals. In the process of coming into contact with water, some free radicals obtain electrons through PVDF, forming PVDF*.

The specific reactions are as follows:

(2) $$H_{2}O\;-e^{-}\;\to {\;H}_{2}O\cdot^{+}$$

(3) $$H_{2}O+H_{2}O\cdot^{+}\;\to {\;H}_{3}O^{+}+\cdot \mathrm{OH}$$

(4) $$\mathrm{PVDF}+{\;e}^{-}\;\to {\mathrm{PVDF}}^{*}$$

(5) $${\mathrm{PVDF}}^{*}-{\;e}^{-}\;\to \mathrm{PVDF}$$

(6) $$O_{2}+{\;e}^{-}\;\to \;\cdot O_{2}^{-}$$

(7) $$\cdot O_{2}^{-}+{\;H}_{3}O^{+}\;\to {\;H}_{2}O+\cdot \mathrm{OH}$$

**Figure 7:**
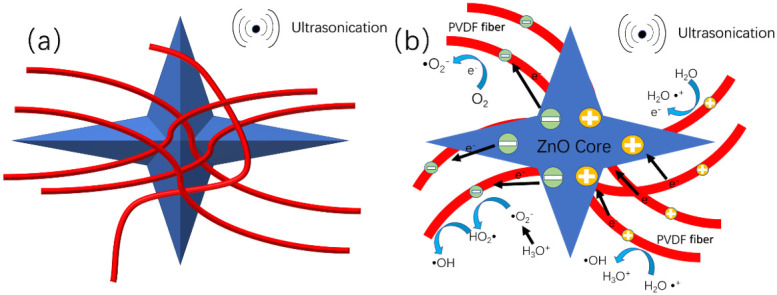
Mechanism of the contact-piezoelectric bi-catalysis of MO degradation under ultrasonication. (**a**) Schematic diagram of the structure of ZnO@PVDF composite membrane (red = PVDF fiber; blue = tetrapodal ZnO). (**b**) Schematic diagram of proposed mechanism of the contact-piezoelectric bi-catalysis.

As shown in Figure 7, when the cavitation bubbles produced by the ultrasonic waves collapse, a high-pressure and high-temperature microjet results, which shocks the previously adsorbed water molecules on the ZnO@PVDF composite membrane. The water loses electrons to form ·$H_{2}O\cdot^{+}$, which reacts with $H_{2}O$ to form hydroxyl radicals ·OH and hydronium ions $H_{3}O^{+}$, One electron is transferred from water to PVDF upon contact, and the notation of PVDF* is proposed to describe the charged state of PVDF after separating from water. At the same time, the O_2_ grabs the electron from the charged surface of PVDF*, which will retrieve its initial uncharged state after transferring this electron to O_2_ to form O_2_^−^ PVDF transfers the electron to the oxygen, and PVDF reverts back to its original form, and PVDF realizes the catalytic effect. The $H_{3}O^{+}$ can react with $\cdot O_{2}^{-}$ to form pure water and hydroxyl radical ·OH. Oxygen dissolved in the water under ultrasonication will exhibit a fierce vibration, impinging on the surface of the PVDF. Oxygen has the ability to attract electrons from the negatively charged PVDF, leading the PVDF to continue to cycle in the charged and uncharged state, and the system continues to produce free radical ·OH, which is the main reactive radical for the degradation of the MO solution.

At the same time, the current carriers in the piezoelectric catalytic process of ZnO and the β-phase of PVDF are mainly electrons and holes, and the ultrasonic wave is one pressure force affecting electron/hole production in ZnO and PVDF (β-phase). We can assume that there are positive and negative charges on the ZnO particles and at the edge of the β-phase of PVDF. While the dielectric PVDF fibers can further separate the piezo-generated electrons/holes and lower the recombination rate. So on the one side, the piezoelectric ZnO particles and the β-phase of PVDF provide more charges spreading to the surface of PVDF composite fibers to accelerate the reaction with MO; on the other hand, the PVDF composite fibers prolong the recombination time because of their ability to store the charge of dielectric material. The synergy between the two compositions prolongs the existence time of the electrons/holes, providing the opportunity to produce hydroxyl radical. Consequently, the degradation efficiency is inevitably improved. Therefore, the reasonable design of catalysts by combining substrate materials with a good contact electric effect and functional materials with a good piezoelectric effect can greatly improve the efficiency of the catalysis, and at same time, providing potential for the catalytic degradation of organic contaminant.

## 3. Materials and Methods

### 3.1. Chemicals

PVDF powder was obtained from Shanghai 3F New Material Co., Ltd. N, N-dimethylformamide, MO and acetone were purchased from Sigma Adrich (Shanghai, China). Tetrapodal ZnO was provided by Qicaiguan (Changzhou, China). Water (resistivity > 18 mΩ cm) was obtained from a Merck Milli-Q Reference system (Darmstadt, Germany). All chemicals are at least reagent grade, and were used without further purification.

### 3.2. Experimental Procedures

#### 3.2.1. Preparation of the ZnO@PVDF Composite Membranes

The PVDF fiber membrane was prepared by electrospinning technology. A homogeneous solution of PVDF (10 wt%) was prepared by dissolving PVDF powder in DMF and acetone (1/1, *v*/*v*), and the mixture was magnetically stirred for 1.5 h at 60 °C in a water bath. All electrospinning fibers were prepared with an electrospinning speed of 1 mL/h, an electrospinning voltage of 15 kV, a collection distance of 15 cm, and a collection rolling speed of 300 rpm. PVDF fibers were collected on aluminum foil to form the electrospinning fiber membrane. The prepared standard sample was marked as pure PVDF. Five different mass ratios of ZnO/PVDF spinning solutions (1:1, 1:2, 1:3, 1:4, 1:5) were prepared using the same method, and the tetrapodal ZnO was completely dispersed in the spinning solution, without delamination. Since ZnO will change the conductivity of the original spinning solution, the voltage was changed slightly according to the proportion of ZnO to meet the spinning demand [31]. The samples were named as 1:1, 1:2, 1:3, 1:4, and 1:5, respectively. 

#### 3.2.2. Catalytic Decomposition of Methyl Orange

In a typical procedure, 5 mg C_14_H_14_N_3_NaO_3_S was added to 1 L distilled water, and the solution was stirred for 1 h to prepare 5 ppm MO aqueous solution. Samples 1:1, 1:2, 1:3, 1:4, 1:5, and pure PVDF were cut into round shapes, with a diameter of 3 cm, and put into a 50 mL beakers labeled as A, B, C, D, E, F, G, respectively. Then, 5 mL of 5 ppm MO solution was added to each beaker under ultrasonication (80 kHz, 700 W) at room temperature. MO solution samples were removed for the UV-Vis spectrum test at an interval of 1.5 h. 

In addition, to explore the effect of MO concentration on degradation efficiency, MO solutions with concentrations of 2 ppm, 5 ppm, 10 ppm, 15 ppm, and 20 ppm, respectively, were prepared using the above method, and the ultrasonic degradation steps were repeated. All experiments were conducted in duplicate.

### 3.3. Characterization and Measurements

Ultrasonic catalysis of the samples was carried out using an ultrasonic cleaning machine (SY-700). The degradation effect of the samples was characterized by absorbance using a UV-Vis spectrum ester (UV-2700, Shimadzu, Kyoto, Japan). The morphology of the samples was characterized by SEM (JSM-6390LV, Japan Electronics Co., Ltd., Tokyo, Japan). The EDS energy spectrum analysis test was applied to determine the element content and distribution on the surface of the samples using JSM-7800F (Nippon Electronics Co., Ltd., Tokyo, Japan) The TG test was performed by thermo-gravimetric analysis using a synchronous thermal analyzer (SDT-650). 

## 4. Conclusions

A novel contact-piezoelectric bi-catalysis of a ZnO@ PVDF composite membrane was prepared using electrospinning technology. The as-prepared ZnO@PVDF composite membrane is superior to the pure PVDF electrospinning membrane in decomposing MO under ultrasonication at room temperature, which is mainly attributed to the synergy effect of the contact-electro-catalysis of dielectric PVDF and the piezoelectric catalysis of ZnO and PVDF (β-phase). The heterostructure of the piezoelec-ZnO@dielectric-PVDF composite interface is helpful in reducing the electron/hole pair recombination. The degradation efficiency of MO solution with 5 ppm was improved to 55.29%, using the ZnO@PVDF composite membrane, from 10.60%, using the pure PVDF membrane, after 360 min under ultrasonication (80 kHz, 700 W). The reusability and stability of the membrane are both comparable to those of the traditional powdered catalyst. After three-cycle degradation, the ZnO content and morphology of the ZnO@PVDF composite membrane changed little. This work suggests one potential strategy for enhancing the catalytic efficiency of dye decomposition by combining contact-electro-catalysis with piezoelectric catalysis. 

## Data Availability

Not applicable.

## Sample Availability

Not available.
